# 713. Prevalence of Microbicide Resistance Genes in Clinical Isolates of *Acinetobacter baumannii*

**DOI:** 10.1093/ofid/ofad500.775

**Published:** 2023-11-27

**Authors:** Piyali Chatterjee, Munok Hwang, Hosoon Choi, Sorabh Dhar, Keith S Kaye, Curtis Donskey, Chetan Jinadatha

**Affiliations:** Central Texas Veterans Health Care System, Temple, Texas; Central Texas Veterans Health Care System, Temple, Texas; Central Texas Veterans Health Care System, Temple, Texas; Wayne State University/Detroit Medical Center, John Dingell VAMC, Detroit, Michigan; Rutgers Robert Wood Johnson Medical School; Cleveland VA Hospital, Cleveland, Ohio; Central Texas Veterans Health Care System, Temple, Texas

## Abstract

**Background:**

The persistence of *Acinetobacter* on different surfaces and the continuous exposure of bacteria to disinfectants such as quaternary ammonium compounds (QACs) in the healthcare environment have led to microbicide tolerance. *Acinetobacter baumannii* have also rapidly acquired carbapenem resistance. Here we determined the prevalence of microbicide resistance genes in carbapenem susceptible and resistant clinical isolates of *Acinetobacter*.

**Methods:**

All clinical isolates were collected from two separate hospitals in Detroit between 2017-2021. Following DNA isolation using Qiagen kit, Nextera Flex kit was used for library preparation of *Acinetobacter* clinical isolates. Whole genome sequencing (WGS) was performed via Illumina NextSeq 550 using the prepared DNA library. The FASTQ files from the sequencing run were then subjected to bioinformatic analysis with Bionumerics software v7.6. ResFinder was used for determination of presence or absence of QAC resistant genes and results were tabulated by Carbapenem-Resistant *Acinetobacter baumannii* (*CRAb*) or Carbapenem-Susceptible *Acinetobacter baumannii* (*CSAb*) with or without the QAC resistant genes.

**Results:**

Out of the 139 *Acinetobacter* patient isolates, 70/139 (50.4%) were *CRAb* containing carbapenemase *bla*OXA-23 and 69/139 (49.6%) belonged to *CSAb* (Table 1). Figure 1. shows the prevalence of antimicrobial resistance (AMR) genes in *CRAb* and *CSAb* isolates with *qac*E genes. For *CRAb* isolates, 56% had *qac*E genes while 39% of *CSAb* had *qac*E genes. About 42% of *CRAb* isolates were multi-drug resistant (MDR), indicating a prevalence of *qacE* genes among the MDR isolates. Table 2 shows the presence of *qacE* genes by sequence type (ST). In the ST2 (Pasteur) and ST406 (Pasteur) strains, *qacE* gene was mainly present. Interestingly, most of the *Acinetobacter* isolates that belong to ST2 (ST195 and ST281, Oxford) are *CRAb* with *qacE* gene.

**Figure:**
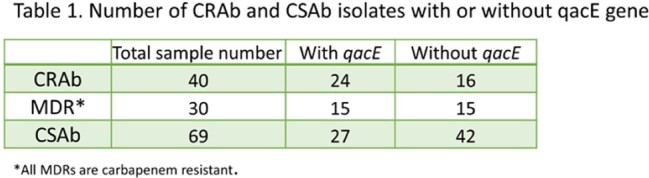

**Figure 1:**
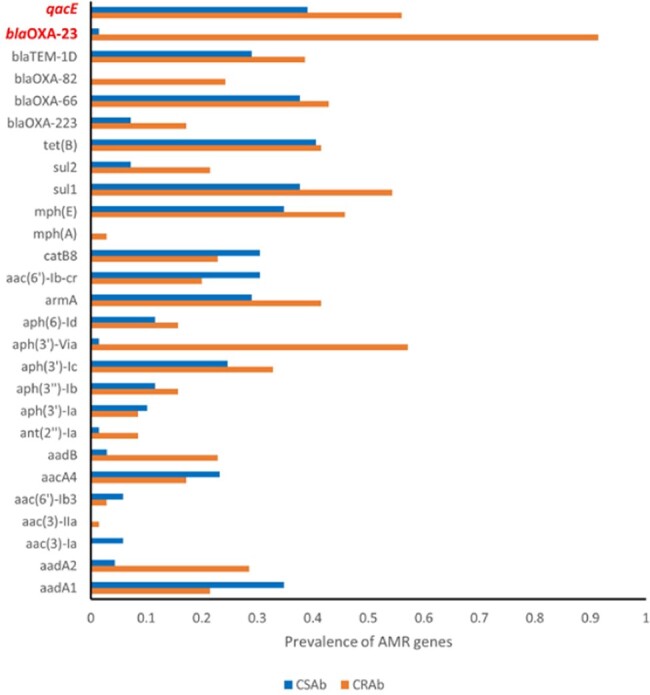
Different AMR genes and qacE in both CRAb and CSAb isolates

**Figure:**
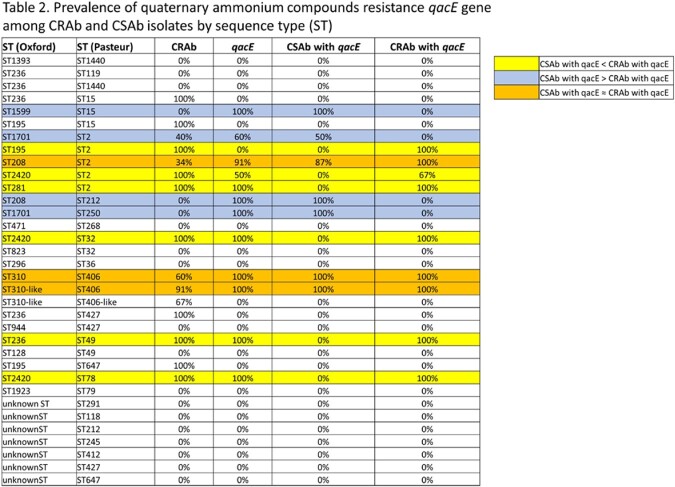

**Conclusion:**

Clinical isolates in our study had an even distribution of both *CRAb* and *CSAb*. The presence of *qacE* gene was more prevalent in the *CRAb* isolates compared to the *CSAb* isolates. While different STs harbored the *qacE* gene, the prevalence of *qacE* gene was highest in ST2 among all isolates. Future studies are needed to determine the reduced susceptibility to other commonly used hospital disinfectants.

**Disclosures:**

**Keith S. Kaye, MD, MPH**, Abbvie: Advisor/Consultant|Abbvie: Honoraria|Entasis: Advisor/Consultant|Entasis: Honoraria|GSK: Advisor/Consultant|GSK: Honoraria|Merck: Advisor/Consultant|Merck: Honoraria|Shionogi: Advisor/Consultant|Shionogi: Honoraria|VenatoRx: Advisor/Consultant|VenatoRx: Honoraria

